# Machine learning study on predicting depressive symptoms and genetic correlations in Parkinson’s disease

**DOI:** 10.3389/fnagi.2025.1584005

**Published:** 2025-04-09

**Authors:** Haijun Zhang, Yifan Zhang, Guihua Li

**Affiliations:** ^1^Department of Neurology, ShenzhenBaoan People’s Hospital, Shenzhen, China; ^2^Department of Neurology, The Affiliated Guangdong Second Provincial General Hospital of Jinan University, Guangzhou, China; ^3^The Second Clinical Medical College of Southern Medical University, Guangzhou, Guangdong, China

**Keywords:** machine learning, clinical prediction, genetic correlations, Parkinson’s disease, depression

## Abstract

Depressive symptoms are prevalent in individuals with Parkinson’s disease. Previous research has demonstrated a significant association between the triglyceride glucose (TyG) index and depression. Leveraging multicenter clinical data, the present study evaluates the predictive capacity of the TyG index for depressive symptoms in PD patients, aiming to establish its potential role in identifying individuals at risk for depression. A comparative analysis of multiple machine learning models was conducted to predict depression in PD patients, ultimately selecting the most effective model. Key predictive variables, including diabetes status, sex, cholesterol levels, triglycerides, blood glucose, and sleep disturbances, were incorporated into a support vector machine (SVM)-based nomogram to assess depression risk in PD patients. Additionally, a genome-wide association study (GWAS) utilizing external databases confirmed a causal relationship between the TyG index and depression. Furthermore, this study explores the biological functions and molecular mechanisms underlying shared transcriptomic proteins between PD and depression, providing insights into potential pathophysiological links between the two conditions.

## 1 Introduction

Motor impairments in Parkinson’s disease (PD) significantly restrict patient mobility, and the resultant slowing of daily activities has a profound impact, particularly on younger individuals with occupational responsibilities. These motor limitations, coupled with reduced physical activity, contribute to an increasing burden of emotional and psychological symptoms, which necessitate growing clinical attention (Xiang et al., 2024). Classical pathways involving dopaminergic, serotonergic, and noradrenergic dysfunctions are implicated in the disruption of neurotransmitters associated with depression (Fu et al., 2020). Interestingly, depression is both a common comorbidity of motor symptoms and may exacerbate these symptoms, but it can also precede the onset of motor dysfunction (DeBroff et al., 2023). Certain genetic mutations in PD, such as those in LRRK2 and GBA, have been shown to increase the likelihood of developing depressive symptoms (Hasuike et al., 2020; Hurh et al., 2022). Thus, understanding the relationship between depression and PD is of significant clinical value for predicting disease progression.

Previous studies have demonstrated that severe depressive symptoms in PD patients are often accompanied by disturbances in glucose and lipid metabolism. Specifically, fasting plasma glucose (FPG) levels have been found to be associated with depression in PD patients (Kalyanaraman et al., 2024). Elevated blood lipid levels, conversely, may be linked to an increased risk of PD. This association could be due to the neuroprotective role of cholesterol precursors in substantia nigra neurons (Jeong et al., 2021; Al-kuraishy et al., 2023; Kwon et al., 2023; Lawlor, 2003; Byeon, 2020). Coenzyme Q10 has also been suggested to exert neuroprotective effects by reducing cholesterol levels, mitigating oxidative stress and inflammation, and regulating cholinergic function (Behnoush et al., 2024). Moreover, overexpression of phospholipid metabolism has been observed in PD patients. In terms of pharmacological interventions, statins, which have anti-inflammatory effects, may offer protection to dopaminergic neurons; however, their lipid-lowering effects may simultaneously increase the risk of PD (Laux, 2022; Sun et al., 2025). Additionally, hyperglycemia and insulin resistance have been associated with a higher incidence of depression (Wan and Yu, 2024; Graham et al., 2017). Genetic correlations between lipid metabolism and depression have been reported, and insulin resistance, as reflected by the triglyceride-glucose (TyG) index, has gained increased attention in recent years for its role in various diseases (Khan et al., 2023). Insulin resistance, as indicated by the TyG index, has been inversely correlated with depression (Borgonovo et al., 2017; Teng et al., 2022; Antar et al., 2021). However, findings regarding gender differences in this relationship remain inconsistent, the aim of this study is to investigate whether the TyG index is associated with depressive symptoms in PD patients and to explore its potential as a predictive marker for both depression and PD risk.

## 2 Methods and methods

### 2.1 Study population

This study was conducted in three parts. The first part comprised a cross-sectional clinical investigation, utilizing retrospective clinical and laboratory data from two cohorts: the Haizhu cohort from Guangdong Second Provincial General Hospital and the Baoan cohort from Shenzhen Baoan People’s Hospital. A total of 300 and 271 complete cases, respectively, were included, comprising PD patients diagnosed between 2013 and 2023. To ensure sufficient statistical power, sample size estimation and power analysis were performed. Based on prior research data, we hypothesized a mean difference of 0.3 in the TyG index between the depression and non-depression groups, with a standard deviation of 0.6. The statistical power was set at 0.8, and the significance level (*α*) was set at 0.05. Using G*Power software, the minimum required sample size to detect this effect size was determined to be 260 patients. The retrospective analysis focused on demographic characteristics, medical history, and laboratory findings, with particular emphasis on blood lipid levels, fasting blood glucose, and the TyG index, which was calculated using established formulas (Haure-Mirande et al., 2019). Depression was assessed using the Hamilton Depression Rating Scale (HAMD), with a score > 10 indicating depression. Based on this threshold, PD patients were categorized into depression (HAMD >10) and non-depression (HAMD ≤10) groups. Sleep disorders were evaluated using the Pittsburgh Sleep Quality Index (PSQI), with a score > 8 serving as the diagnostic criterion (Mollayeva et al., 2016).

The second and third parts of the study employed publicly available datasets to investigate the association between PD and depression using Mendelian randomization (MR) analysis. The exposure variables for the MR analysis included 192 single-nucleotide polymorphisms (SNPs) significantly associated with the TyG index (*p* < 5 × 10^−8) from prior studies, while the outcome variables were derived from depression-related data in the UK Biobank. Covariates included age, sex, stroke, diabetes, sleep disorders, triglycerides (TG), and blood glucose. Furthermore, to assess and control for the potential influence of horizontal pleiotropy on causal inference, we conducted sensitivity analyses using MR-Egger regression, weighted median estimation, and MR-PRESSO. These methods were employed to ensure that the instrumental variable influenced depression exclusively through the TyG index, rather than through alternative pathways. Data with continuous imbalance were excluded, and a two-sample Inverse variance weighted regression analysis was performed.

**Figure 1:**
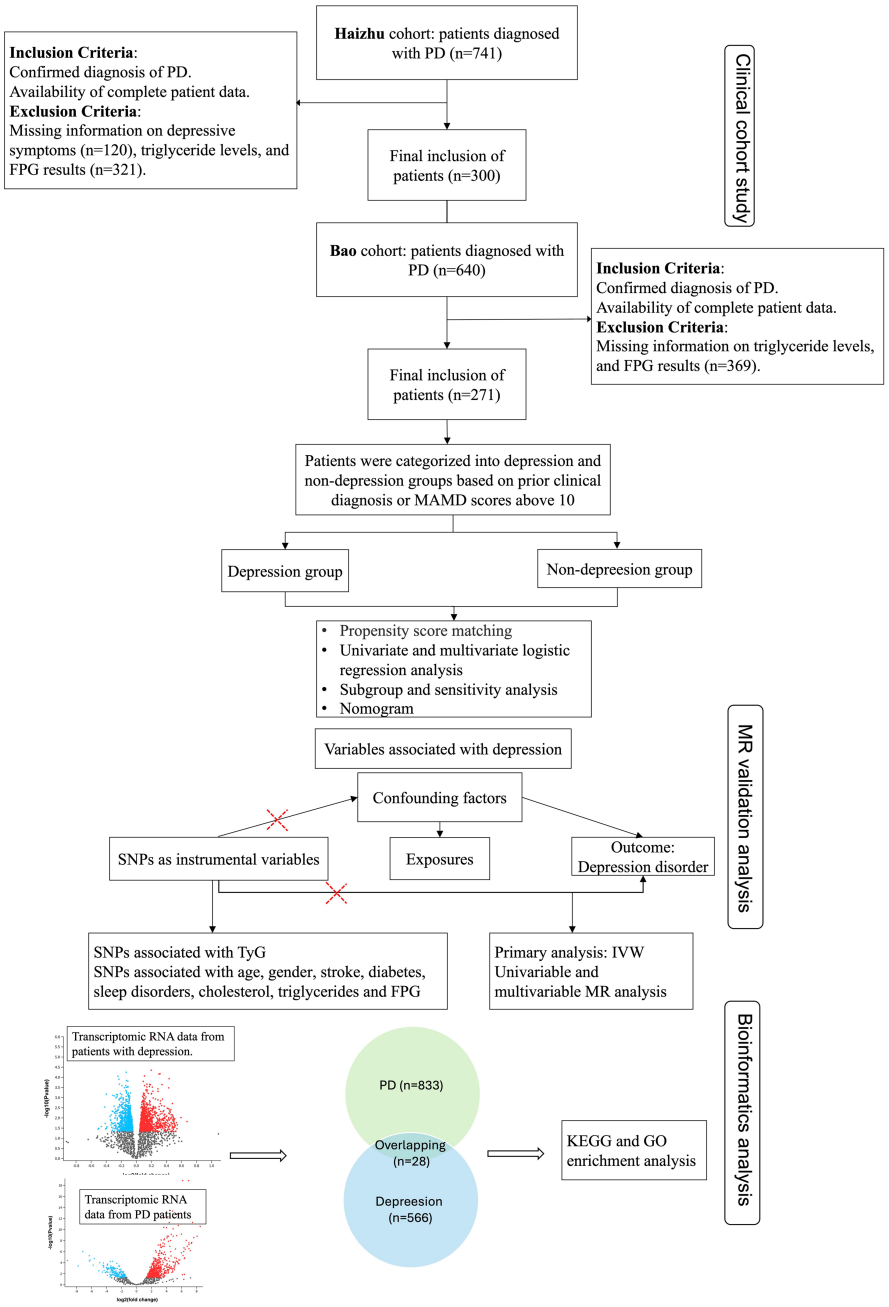
Flowchart of this study. CAD, Coronary artery disease; PD, Parkinson’s disease; HAMD, Hamilton Depression Rating Scale; FPG, Fasting plasma glucose; KEGG, Kyoto encyclopedia of genes and genomes; GO, Gene ontology; IVW, Inverse variance weighted.

Furthermore, differential expression analyses of transcriptional RNA in peripheral plasma samples from patients with PD and depression were performed using the datasets GSE160299 and GSE39653. This analysis identified 28 overlapping genes. These shared proteins were subsequently subjected to functional enrichment analyses, including STRING protein–protein interaction networks, as well as KEGG and GO pathway analyses, to elucidate common molecular mechanisms underlying both conditions. All data were obtained from publicly available databases, with detailed sources of GWAS data provided in Supplementary Table 1 (The flowchart is shown in Figure 1).

### 2.2 Statistical analysis

Categorical variables were compared using chi-square tests, while continuous variables were compared with the Mann–Whitney U test. To minimize potential confounding effects and ensure comparability between groups, propensity score matching (PSM) was applied for baseline data adjustment. Logistic regression analysis was conducted to identify potential factors associated with depressive outcomes, and subgroup analyses were conducted to evaluate the impact of the TyG index across different patient subpopulations. To enhance predictive accuracy, multiple machine learning models were employed, including logistic regression, random forest, support vector machine (SVM), and extreme gradient boosting (XGBoost). Model performance was assessed using key evaluation metrics, including accuracy, precision, recall, F1-score, and the area under the receiver operating characteristic curve (AUC), allowing for a comprehensive comparison of their effectiveness in predicting depression risk in PD patients. In addition, a clinical prediction nomogram was constructed using various selected variables, and receiver operating characteristic (ROC) curves were plotted to compare the AUC values of different models. Causal MR analysis of the TyG index and depression was performed using odds ratios (OR) and 95% confidence intervals (CI), with heterogeneity considered and sensitivity analyses conducted to adjust for potential confounding factors. Differentially expressed genes identified between PD, depression, and control groups were further analyzed to identify overlapping miRNAs as potential key regulators. Protein–protein interactions were explored using the STRING online tool and Cytoscape. All other statistical analyses were carried out using R version 4.4.2.

## 3 Results

**Figure 2:**
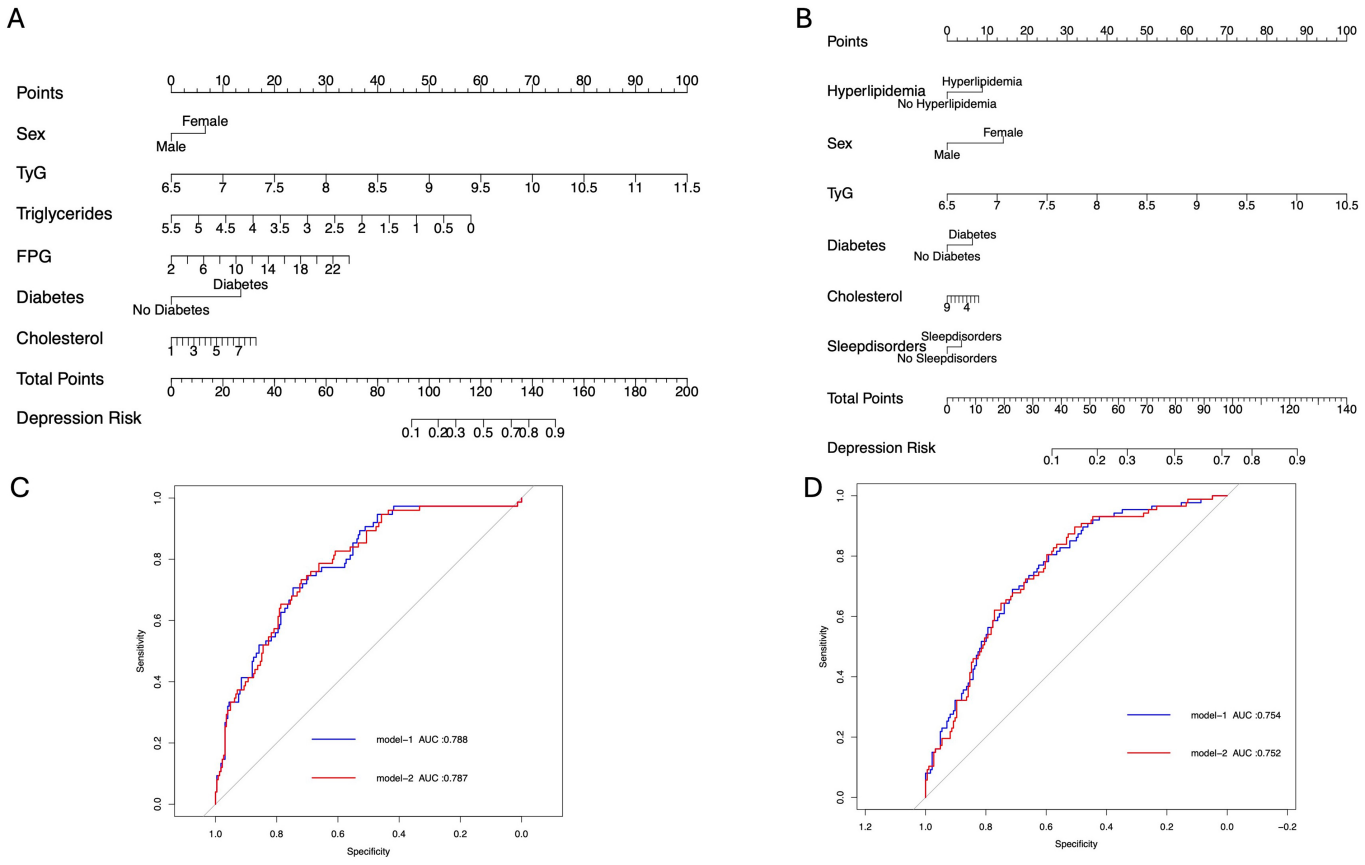
Clinical prediction model diagram. **(A,B)** Nomograms of Model 1 for predicting depression occurrence in the Haizhu and Baoan cohorts. **(C,D)** ROC curves of Models 1 and 2 for predicting depression in the Haizhu and Baoan cohorts.

**Figure 3:**
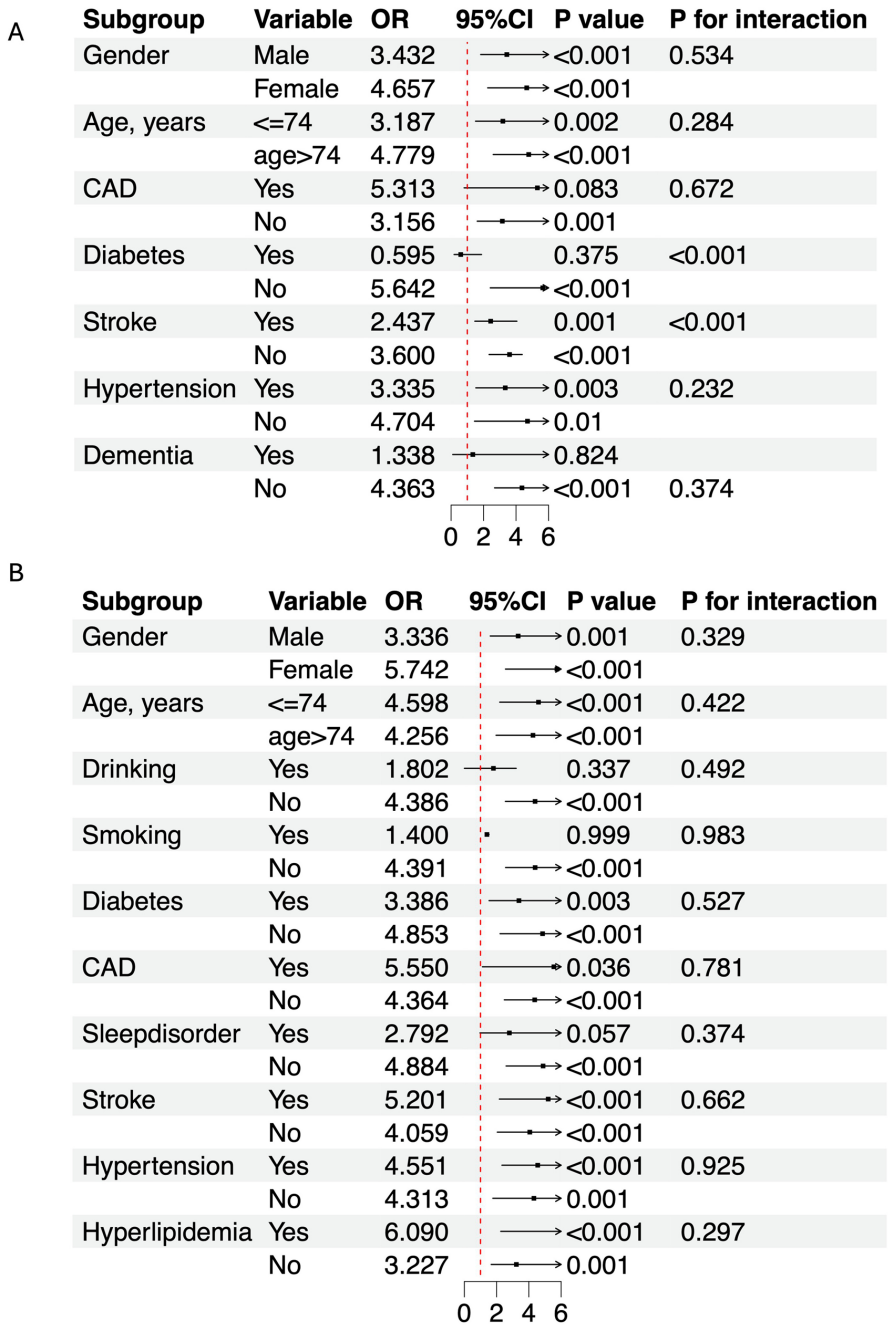
Subgroup analysis of TyG index and depression in PD population. **(A)** Haizhu cohort. **(B)** Baoan cohort. OR, Odds ratio; CI, Confidence interval; CAD, Coronary artery disease.

In this study, 300 PD patients from the Haizhu cohort and 271 from the Baoan cohort were included. In the Haizhu cohort, 75 patients were diagnosed with depression, while 225 did not have depression. In the Baoan cohort, 87 patients were in the depression group, and 184 were in the non-depression group. Before propensity score matching, the depression group showed a higher prevalence of diabetes, female sex, sleep disorders, and hyperlipidemia. After matching, clinical and laboratory variables were better balanced between the two groups. However, the depression group still exhibited a significantly higher TyG index (Table 1). Regression analysis revealed that the TyG index was a key predictor of depressive symptoms in both cohorts. Multivariate analysis, which adjusted for diabetes, sex, cholesterol, triglycerides, blood glucose, and sleep disorders, confirmed that these factors were independent risk factors for depression (Table 2). Machine learning model comparisons (Supplementary Table 1) revealed that XGBoost outperformed other models across both cohorts, achieving the highest accuracy, F1-score, and recall. Random forest also demonstrated strong predictive performance, albeit slightly inferior to XGBoost, but exhibited greater stability compared to logistic regression and support vector machine (SVM). To mitigate the risk of overfitting, random forest was selected for constructing the nomogram, which demonstrated moderate predictive performance with an area under the curve (AUC) ranging from 0.75 to 0.78. Based on these findings, a nomogram incorporating these predictive variables was developed to assess the risk of depressive symptoms in PD patients (Figure 2). Furthermore, subgroup analyses were conducted based on gender, age, coronary artery disease (CAD), diabetes, stroke, hypertension, cognitive impairment, smoking status, sleep disorders, and hyperlipidemia history. The interactions between the TyG index and depression risk, as well as between the TyG index and various phenotypes, were consistent across all subgroups (Figure 3).

**Table 1:** Baseline characteristics of patients with and without depression among participants in the Haizhu and Baoan cohorts, before and after propensity score matching (PSM).

Haizhu cohort	Original data			Propensity score matching		
Characteristic	No-depression (*n* = 225)	Depression (*n* = 75)	*p* value	No-depression (*n* = 75)	Depression (*n* = 75)	*p* value
Demographics						
Age (years)	79 [72, 86]	77 [73, 85]	0.718	79.11 ± 10.92	78.85 ± 8.55	0.875
Gender (Male)	140 (62.2%)	34 (45.3%)	0.015	36 (48%)	34 (45%)	0.870
Medical history						
Hypertension, *n* (%)	161 (71.6%)	55 (73.3%)	0.882	55 (73%)	55 (73%)	1.000
CAD, *n* (%)	50 (22.3%)	19 (25.3%)	0.635	26 (35%)	19 (25%)	0.285
Stroke, *n* (%)	136 (60.4%)	52 (69.3%)	0.215	49 (65%)	52 (69%)	0.728
Diabetes, *n* (%)	50 (22.2%)	33 (44%)	< 0.001	24 (32%)	33 (44%)	0.178
Dementia, *n* (%)	32 (14.6%)	16 (21.3%)	0.203	11 (15%)	11 (15%)	1.000
Laboratory tests						
Cholesterol (mmol/L)	3.97 [3.33, 4.67]	4.3 [3.66, 5.12]	0.005	3.91 [3.5, 4.33]	4.3 [3.66, 5.12]	0.002
HDL-C (mmol/L)	1.1 [0.93, 1.33]	1.14 [0.95, 1.52]	0.145	1.15 [0.96, 1.31]	1.25 [0.98, 1.42]	0.197
LDL-C (mmol/L)	2.25 [1.64,2.9]	2.63 [1.863,3.22]	0.017	2.22 [1.75, 2.7]	2.63 [1.88, 3.15]	0.015
Triglycerides (mmol/L)	0.93 [0.73, 1.31]	1.31 [0.87, 1.73]	< 0.001	0.89 [0.74, 1.29]	1.31 [0.87, 1.73]	0.004
FPG (mmol/L)	5.17 [4.56, 6.03]	6.65 [5.29, 8.39]	< 0.001	5.65 [4.86, 6.43]	6.65 [5.29, 8.39]	0.001
TyG	8.27 [7.97, 8.73]	8.91 [8.48, 9.21]	< 0.001	8.41 ± 0.61	8.81 ± 0.71	< 0.001
Uric acid (umol/L)	335 [261, 395]	341 [263, 395.5]	0.707	327 [254.5, 392.5]	341 [263, 395.5]	0.409
WBC (10^9^/L)	7.2 [5.79, 8.98]	6.73 [5.67, 8.61]	0.289	7.84 [6.48, 8.73]	7.67 [6.06, 7.94]	0.052
Platelets (10^9^/L)	224.5 [190.75, 268.25]	243 [206, 282.25]	0.054	232.5 [208.5, 257.5]	253.2 [213, 276.5]	0.111

Baoan Cohort	Original data			Propensity score matching		
Characteristic	No-depression (*n* = 184)	Depression (*n* = 87)	*P* value	No-depression (*n* = 87)	Depression (*n* = 87)	*P* value
Demographics						
Age (years)	74 [67, 81]	72 [64, 76.5]	0.059	75 [67, 81]	72 [64, 76.75]	0.082
Gender (Male)	114 (61.9%)	37 (42.3%)	0.004	54 (62%)	38 (44%)	0.023
Habits						
Drinking, *n* (%)	11 (6.0%)	0 (0)	0.067	5 (6%)	1 (1%)	0.211
Smoking, *n* (%)	8 (4.3%)	1 (1.1%)	0.279	4 (5%)	1 (1%)	0.368
Medical history						
Hypertension, *n* (%)	101 (54.9%)	53 (60.9%)	0.421	54 (62%)	53 (61%)	0.923
CAD, *n* (%)	19 (10.3%)	11 (12.6%)	0.621	14 (16%)	12 (14%)	0.832
Stroke, *n* (%)	65 (35.3%)	36 (41.4%)	0.408	37 (43%)	36 (41%)	0.945
Diabetes, *n* (%)	50 (27.2%)	35 (40.2%)	0.043	25 (29%)	35 (40%)	0.151
Hyperlipidemia, *n* (%)	35 (19.0%)	32 (36.8%)	0.003	22 (25%)	30 (34%)	0.246
Sleep disorders, *n* (%)	10 (5.4%)	12 (13.8%)	0.035	4 (5%)	12 (14%)	0.066
Laboratory tests						
Cholesterol, (mmol/L)	3.86 [3.18, 4.63]	4.14 [3.56,4,9]	0.067	4.01 ± 1.02	4.2 ± 1.14	0.252
HDL, (mmol/L)	1.16 [0.97, 1.38]	1.24 [1.07, 1.45]	0.062	1.21 ± 0.32	1.27 ± 0.34	0.221
LDL, (mmol/L)	2.390 [1.803, 3.06]	2.690 [2.14, 3.204]	0.015	2.44 ± 0.95	2.71 ± 0.91	0.061
Triglycerides, (mmol/L)	0.88 [0.7, 1.27]	1.1 [0.81, 1.56]	0.002	0.91 [0.71, 1.31]	1.1 [0.81, 1.56]	0.030
FPG, (mmol/L)	5.19 [4.64, 6.28]	5.36 [4.66, 6.47]	0.675	5.09 [4.64, 6.14]	5.36 [4.66, 6.47]	0.555
TyG	8.22 [7.95, 8.63]	8.52 [8.18, 8.79]	< 0.001	8.23 [8, 8.64]	8.52 [8.18, 8.79]	0.006
Uric acid (umol/L)	303.4 [235.5, 373.68]	286.8 [228.4, 360.85]	0.731	315.06 ± 110.13	304.32 ± 104.18	0.510
WBC (10^9^/L)	6.49 [5.51, 8.13]	6.4 [5.56, 7.67]	0.695	6.79 [5.62, 7.97]	6.4 [5.56, 7.67]	0.599
Platelets (10^9^/L)	212.0 [179, 262]	220.0 [177, 264.3]	0.87	221 [176.5, 272.5]	225 [181, 261.5]	0.971

For continuous variable “[],” the values represent quartiles, and for categorical variables “()” indicate percentages. CAD, Coronary artery disease; HDL, High-density lipoprotein; LDL, Low-density lipoprotein; FPG, Fasting plasma glucose; TyG, Triglyceride-glucose Index; BMI, Body mass index; WBC, White blood cells.

**Table 2:** Univariate and multivariate logistic regression analyses for predicting depressive symptoms in Parkinson’s disease patients from the Haizhu and Baoan cohorts.

Haizhu Cohort						
Variable	Univariate analysis		Multivariate Analysis			
			Model 1^a^		Model 2^b^	
	OR (95% CI)	*P* value	OR 95% CI	*P* value	OR (95% CI)	*P* value
Demographics						
Age (years)	1 (0.97–1.02)	0.84				
Gender (Male)	0.56 (0.33–0.95)	0.031	0.595 (0.321–1.103)	0.099	0.543 (0.299–0.986)	0.045
Medical history						
Hypertension	0.94 (0.53–1.69)	0.831				
CAD	0.85 (0.45–1.53)	0.595				
Stroke	0.72 (0.43–1.23)	0.232				
Diabetes	3.98 (2.29–6.97)	<0.001	2.894 (1.556–5.383)	0.001	2.882 (1.551–5.357)	0.001
Dementia	0.65 (0.1–2.58)	0.581				
Laboratory tests						
Cholesterol (mmol/L)	1.39 (1.1–1.77)	0.006	1.188 (0.883–1.599)	0.255		
Triglycerides (mmol/L)	2.15 (1.5–3.19)	<0.001	0.435 (0.138–1.373)	0.156	0.438 (0.143–1.342)	0.149
FPG (mmol/L)	1.39 (1.23–1.6)	<0.001	1.132 (0.87–1.471)	0.356	1.118 (0.869–1.440)	0.385
TyG	4.08 (2.59–6.66)	<0.001	4.842 (0.881–26.594)	0.070	5.415 (1.039–28.231)	0.045
Uric acid (umol/L)	1.01 (0.99–1.01)	0.277				

Baoan cohort						
Variable	Univariate analysis		Multivariate analysis			
			Model 1^c^		Model 2^d^	
	OR 95% CI	*P* value	OR 95% CI	*P* value	OR 95% CI	*P* value
Demographics						
Age (years)	0.97 (0.95–0.99)	0.015				
Gender (Male)	0.45 (0.27–0.76)	0.003	0.488 (0.272–0.876)	0.016	0.470 (0.263–0.840)	0.011
Habits						
Drinking	0.17 (0.01–0.87)	0.087				
Smoking	0.26 (0.01–1.43)	0.202				
Medical history						
Hypertension	1.48 (0.88–2.51)	0.145				
CAD	1.12 (0.5–2.41)	0.77				
Stroke	1.49 (0.88–2.51)	0.134				
Diabetes	2.26 (1.32–3.88)	0.003	1.382 (0.739–2.587)	0.311	1.327 (0.715–2.466)	0.370
Hyperlipidemia	2.48 (1.4–4.39)	0.002	1.567 (0.810–3.033)	0.182		
Sleep disorders	2.78 (1.15–6.87)	0.023	1.201 (0.399–3.620)	0.745	1.280 (0.430–3.812)	0.657
Laboratory tests						
Cholesterol (mmol/L)	1.26 (1–1.61)	0.056	0.951 (0.720–1.256)	0.723	0.978 (0.743–1.287)	0.873
Triglycerides (mmol/L)	3.95 (2.36–4.5)	<0.001				
FPG (mmol/L)	1.09 (1–1.2)	0.067				
TyG	4.44 (2.67–7.75)	<0.001	3.592 (1.953–6.607)	<0.001	3.899 (2.140–7.106)	<0.001
Uric acid (umol/L)	1.1 (1–1.3)	0.737				

^a^Model 1 (Haizhu cohort): Adjusted for gender, diabetes, cholesterol, FPG, triglycerides and TyG index; ^b^Model 2 (Haizhu cohort): Adjusted for gender, diabetes, FPG, triglycerides and TyG index; ^c^Model 1 (Baoan cohort): Adjusted for hyperlipidemia, gender, diabetes, cholesterol, sleepdisorders and TyG index; ^d^Model 2 (Baoan cohort): Adjusted for gender, diabetes, cholesterol, sleepdisorders and TyG index. TyG index. CAD, Coronary artery disease; FPG, Fasting plasma glucose; TyG, Triglyceride-glucose index.

**Figure 4:**
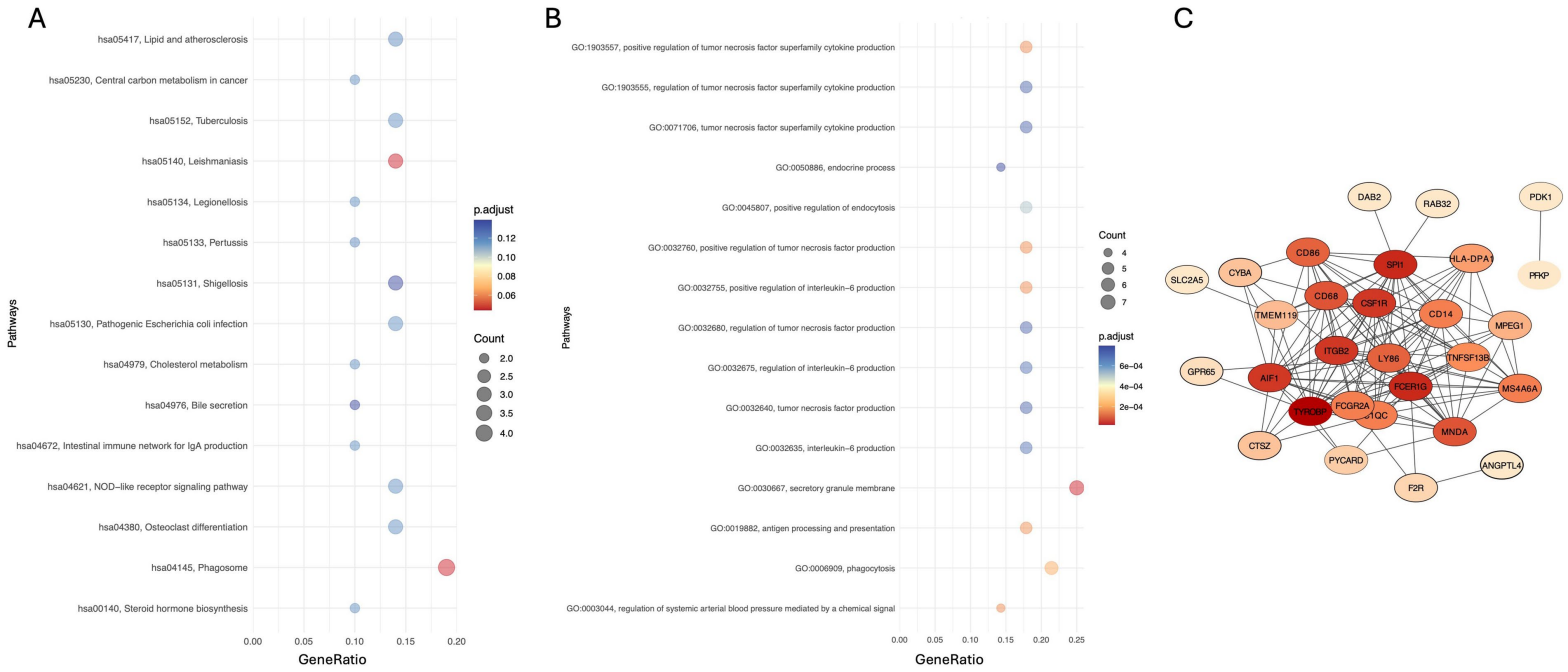
**(A,B)** KEGG and GO enrichment analyses showing the top 15 pathways of differentially expressed genes (DEGs), respectively. **(C)** Protein–protein interaction (PPI) network of DEGs, where yellow nodes represent upregulated genes, and blue and purple nodes represent downregulated genes.

Bidirectional MR analyses revealed a significant association between the TyG index and depression (Beta = 0.011, *p* < 0.001, OR = 1.011). In multivariable MR analysis, after adjusting for potential confounders including age, sex, stroke, diabetes, sleep disorders, cholesterol levels, triglycerides, and FPG, the association between the TyG index and depression remained significant, with the OR ranging from 1.006 to 1.031. Further adjustment for all covariates continued to show a significant association (Beta = 0.015, OR = 1.015, *p* < 0.001) (Table 3). In addition, a total of 833 upregulated genes associated with PD were identified from the GSE160299 dataset, while 566 differentially expressed genes were extracted from the depression-related dataset GSE39653. Intersection analysis of these two datasets, followed by KEGG pathway analysis, revealed that these genes were significantly enriched in pathways related to “gut immune network for IgA production” and “cholesterol metabolism.” Gene Ontology (GO) biological process (BP) analysis indicated substantial enrichment in pathways involved in “chemical signal regulation,” “endocrine processes,” and “interleukin signaling.” Regarding cellular components (CC), the genes were primarily associated with the “secretory granule membrane.” A Protein–Protein Interaction (PPI) network consisting of 28 differentially expressed genes (28 nodes and 136 edges) was constructed, identifying key genes, including TYROBP, SPI1, FCER1G, ITGB2, and CSF1R, which play central roles in the shared molecular mechanisms of depression and Parkinson’s disease (Figure 4).

**Table 3:** Causal association between the TyG index and depression through bidirectional and multivariable Mendelian randomization analyses.

Models	Method	nsnp	Beta	Se	*p* value	OR 95%CI
Crude analysis						
TyG	IVW	96	0.011	0.003	<0.001	1.011 (1.005–1.016)
Sensitivity analysis						
Adjusted for age	IVW	16	0.028	0.010	0.006	1.028 (1.008–1.048)
Adjusted for gender	IVW	31	0.018	0.008	0.019	1.018 (1.003–1.033)
Adjusted for stroke	IVW	32	0.027	0.006	<0.001	1.027 (1.015–1.039)
Adjusted for diabetes	IVW	22	0.031	0.008	<0.001	1.031 (1.015–1.048)
Adjusted for sleep disorders	IVW	24	0.028	0.008	<0.001	1.029 (1.014–1.044)
Adjusted for cholesterol	IVW	15	0.026	0.009	0.006	1.026 (1.007–1.045)
Adjusted for triglycerides	IVW	14	0.006	0.002	0.008	1.006 (1.002–1.010)
Adjusted for FPG	IVW	13	0.022	0.008	0.005	1.022 (1.006–1.038)
Sensitivity analysis						
Adjusted for age, gender, stroke, diabetes, sleep disorders, cholesterol, triglycerides and FPG						
TyG	IVW	61	0.015	0.003	<0.001	1.015 (1.010–1.021)

FPG, Fasting plasma glucose; TyG, Triglyceride-glucose index.

## 4 Discussion

This study, utilizing retrospective clinical data from a two-center cohort, investigates the role of the TyG index in predicting depressive symptoms in PD. A clinical prediction model was developed incorporating factors such as gender, age, history of diabetes, and other relevant variables. Subgroup analysis revealed the impact of confounding factors on the relationship between the TyG index and depressive symptoms in PD. MR analysis further confirmed that an elevated TyG index increases the risk of depression. Moreover, the identification of peripheral blood plasma proteins involved in shared molecular pathways for PD and depression provides valuable insights into potential signaling mechanisms. Despite the implementation of bidirectional and sensitivity MR analyses, several limitations must be considered, including potential population stratification bias, gene–environment interactions, and the influence of lifestyle factors on the relationship between the TyG index and depression risk.

### 4.1 Predictive role of the TyG index in PD-associated depression

The positive association between the TyG index and depression risk has been corroborated by several studies, consistently observed across different age groups, genders, and individuals with other chronic conditions (Hong et al., 2021; Meng et al., 2020; Jiang et al., 2024). The effect of diabetes on the TyG index is multifactorial, extending beyond insulin resistance. For instance, antidiabetic medications such as SGLT2 inhibitors have been shown to influence the TyG index (Shi et al., 2021; Shimoda et al., 2023; Wan and Yu, 2024). In our study, the predictive value of the TyG index for depression was notably reduced in the diabetic subgroup, likely due to the influence of pharmacological interventions. Furthermore, older individuals with more severe depressive symptoms are at a higher risk of developing diabetes (Graham et al., 2017), which aligns with our finding of a higher proportion of diabetic patients among those with depression.

Our study population comprised PD patients, particularly older cohorts, making age a potentially significant factor influencing the results. Research suggests that the TyG index may be involved in the physiological processes of diabetes and vitamin D deficiency in the elderly (Xiang et al., 2024), potentially exacerbating the burden of cognitive impairment (Teng et al., 2022), with vitamin D supplementation being linked to the alleviation of depressive symptoms (Casseb et al., 2019). In the Haizhu cohort, we did not find significant differences in cognitive impairment between depressed and non-depressed PD patients, although studies have shown that PD patients with apathy and anhedonia may exhibit more severe cognitive impairment (Santangelo et al., 2009). Additionally, patients with more severe cognitive deficits may be at increased risk of developing depression or worsening depressive symptoms (Koros et al., 2023). Zheng et al.’s study, which included middle-aged and elderly patients with a mean age of 58, supported the TyG index as an independent factor influencing depression (Zheng et al., 2023). However, there is currently insufficient evidence to suggest that the TyG index can independently affect depressive symptoms in elderly patients. Therefore, when considering depressive symptoms in elderly PD patients, a comprehensive assessment of the TyG index in conjunction with other factors, such as pharmacological interventions, vitamin D levels, cognitive function status, and other potential biomarkers, is necessary to fully understand the complexity of depression.

### 4.2 Clinical predictive assessment

Compared to previous predictive models, which primarily relied on psychological scales (Camerucci et al., 2024; Byeon, 2020), serum and cerebrospinal fluid neurofilament light chain (NfL) levels (Li et al., 2021; Urso et al., 2023), and radiomics-based approaches for predicting depressive symptoms and other non-motor symptoms in PD (Antar et al., 2021), this study introduces a novel approach by incorporating diabetes- and lipid-related clinical variables. Additionally, previous studies have suggested that REM sleep behavior disorder (RBD) and olfactory dysfunction may serve as potential longitudinal predictors of PD. A key strength of this study lies in the selection of readily accessible clinical variables, enhancing the model’s practicality for real-world application. Moreover, the use of multicenter data improves the generalizability and interpretability of the predictive model. However, there is room for improvement in terms of data diversity and the analysis of interactions among predictive variables, which could further refine the model’s predictive accuracy and robustness.

### 4.3 Biological mechanisms underlying PD-associated depression

This study underscores the intricate relationship between lipid metabolism dysregulation, inflammatory signaling, and their roles in the pathophysiology of PD and depression. *α*-Synuclein aggregation has been shown to induce abnormal lipid accumulation (Kamano et al., 2024), which subsequently disrupts lipid metabolism and further impairs mitochondrial function. Additionally, alterations in mitochondrial gene expression, along with damage to mitochondrial membrane proteins and lipids, are observed in the central nervous system of patients with depression (Khan et al., 2023).

The transcriptomic analysis in this study identified core overlapping genes between PD and depression, suggesting the presence of shared immune-inflammatory pathways in their pathogenesis. Previous research has demonstrated that TYROBP plays a crucial role in microglial inflammatory activation and the maintenance of neuronal microenvironment stability, with its dysregulated expression potentially contributing to the onset and progression of PD (Haure-Mirande et al., 2019). Additionally, SPI1 has been identified as a shared gene between PD and major depressive disorder (Wang et al., 2023), while ITGB2 is upregulated in patients with unipolar depression (Dmitrzak-Weglarz et al., 2021). These genes are closely associated with neuroinflammation and immune regulation and may influence PD and depression pathogenesis by modulating the activation of microglia and peripheral immune cells. Enrichment analysis further highlighted the gut immune network for IgA production as a potentially critical pathway underlying PD-related depression. Alterations in gut microbiota have been implicated in various non-motor symptoms of PD, with dysbiosis potentially preceding the onset of motor symptoms. Previous studies have reported a reduction in IgA-associated microbiota in PD patients, which may disrupt gut microbial homeostasis and exacerbate neuroinflammation (Brown et al., 2023). Furthermore, IgA deficiency could contribute to greater gut microbiota dysregulation, leading to the release of pro-inflammatory cytokines and potentially playing a role in the neuroinflammatory mechanisms underlying inflammation-related depression (Saha et al., 2022; Liu et al., 2024). Understanding the relationship between gut microbiota and PD-related depression is essential for elucidating the role of the neuroimmune-gut axis in the development of depressive symptoms in PD. Gut dysbiosis may influence the progression of PD-associated depression through multiple mechanisms, including the regulation of short-chain fatty acid (SCFA) production, modulation of neurotransmitter levels within the gut-brain axis, mediation of systemic inflammatory responses, and regulation of intestinal barrier function (Kalyanaraman et al., 2024).

The transcriptomic and proteomic associations between the TyG index, depression, and PD reflect the intricate interplay between glucose-lipid metabolism and immune regulation. An elevated TyG index is indicative of insulin resistance, and dysregulation of the insulin signaling pathway may contribute to blood–brain barrier (BBB) endothelial alterations by affecting tight junction proteins, thereby increasing BBB permeability (Rhea and Banks, 2019). Glucose metabolism within BBB endothelial cells relies on the highly coordinated interactions among endothelial cells, pericytes, and astrocytes. When BBB integrity is compromised, immune-related signaling pathways are activated, leading to the release of pro-inflammatory cytokines (Zhu et al., 2025), this inflammatory cascade exacerbates neuroinflammatory responses in both PD and depression, potentially contributing to disease progression.

This study still has several limitations. First, maintaining consistency between clinical cross-sectional data and publicly available datasets presents a methodological challenge. The retrospective clinical data in this study were primarily derived from an Asian population, providing a practical and accessible reference for individualized risk assessment in PD patients. In contrast, GWAS data, predominantly from European populations, emphasize genetic risk factors and leverage large sample sizes, allowing for a more precise evaluation of depression risk in PD at the genetic level. Additionally, transcriptomic data offer insights into RNA expression patterns, facilitating the identification of shared gene regulatory networks between PD and depression. Despite differences in methodology and population sources, these datasets collectively contribute to the prediction of depressive symptoms in PD and the exploration of underlying pathophysiological mechanisms. Second, the cross-sectional design of this study limits its ability to capture the temporal dynamics between the TyG index and PD-related depression. The lack of control over time-dependent effects and potential confounding variables poses challenges in establishing causal inferences. Moreover, the absence of longitudinal follow-up data restricts a comprehensive understanding of disease progression, which may affect the accuracy and generalizability of the predictive model. Despite these limitations, the findings highlight the potential of the TyG index as a predictive biomarker for depression risk in PD patients. Furthermore, variables such as sex and diabetes status have been identified as contributing factors to depression susceptibility. From a mechanistic perspective, dysregulation in lipid metabolism and immune-inflammatory pathways appears to play a central role in the pathogenesis and progression of both PD and depression.

### 4.4 Clinical applications and limitations

The clinical implications of this study suggest that an elevated TyG index may indicate a higher risk of depression in patients with PD. This finding underscores the importance of psychiatric and psychological assessments for PD patients with increased TyG levels, enabling early detection of depressive symptoms and timely clinical intervention. However, the stability of the TyG index may be influenced by individual metabolic factors, including dietary habits, medication use (e.g., lipid-lowering and glucose-lowering drugs), age, and lifestyle, which could introduce variability in its predictive capacity. Additionally, the predictive efficacy of this model requires further validation through large-scale, multicenter prospective longitudinal studies to ensure its broad applicability and specificity in diverse populations. Furthermore, interventions for depression in PD should not be limited to a single metabolic indicator but should integrate a comprehensive, multidimensional approach incorporating genomics, neuroimaging, psychological assessments, neurobiological markers, and lifestyle modifications. Future research should further investigate the biological mechanisms underlying the association between the TyG index and depression in PD to refine predictive strategies and optimize personalized treatment approaches.

## Data Availability

The datasets presented in this study can be found in online repositories. The names of the repository/repositories and accession number(s) can be found in the article/Supplementary material. All original data for this study have been uploaded to Jianguoyun: https://www.jianguoyun.com/p/DYf0saMQ7f2hDRiT6OwFIAA.
