# Supplementary material for: Machine learning study on predicting depressive symptoms and genetic correlations in Parkinson’s disease
Source: Front Aging Neurosci. 2025 Apr 9;17:1584005. doi: 10.3389/fnagi.2025.1584005 (PMC12014618; doi:10.3389/fnagi.2025.1584005)
Supplement: Supplementary file 1 [file Table_1.DOCX]

**Supplemental table1. Summary of GWAS data for exposure and outcomes and TyG Index-associated SNP Data**

|  |  | **Detailed Information of GWAS Data** | |  |
| --- | --- | --- | --- | --- |
| **Exposure** |  | [GWAS ID](https://gwas.mrcieu.ac.uk/datasets/?gwas_id__icontains=ebi-a-GCST90000050&year__iexact=&trait__icontains=&consortium__icontains=&sort=-gwas_id) |  |  |
|  | age | [ebi-a-GCST90000050](https://gwas.mrcieu.ac.uk/datasets/ebi-a-GCST90000050/) | [Sample size](https://gwas.mrcieu.ac.uk/datasets/?gwas_id__icontains=ebi-a-GCST90000050&year__iexact=&trait__icontains=&consortium__icontains=&sort=sample_size) | [Number of SNPs](https://gwas.mrcieu.ac.uk/datasets/?gwas_id__icontains=ebi-a-GCST90000050&year__iexact=&trait__icontains=&consortium__icontains=&sort=nsnp) |
|  | sex | [ebi-a-GCST90013474](https://gwas.mrcieu.ac.uk/datasets/ebi-a-GCST90013474/) | 542,901 | 9,702,772 |
|  | diabetes | [ebi-a-GCST006867](https://gwas.mrcieu.ac.uk/datasets/ebi-a-GCST006867/) | 452,302 | 25,070,139 |
|  | Sleeplessness | [ukb-b-3957](https://gwas.mrcieu.ac.uk/datasets/ukb-b-3957/) | 655,666 | 5,030,727 |
|  | stroke | [ebi-a-GCST90038613](https://gwas.mrcieu.ac.uk/datasets/ebi-a-GCST90038613/) | 484,598 | 9,587,836 |
|  | TG | [ieu-b-111](https://gwas.mrcieu.ac.uk/datasets/ieu-b-111/) | 441,016 | 12,321,875 |
|  | cho | [ebi-a-GCST90038690](https://gwas.mrcieu.ac.uk/datasets/ebi-a-GCST90038690/) | 484,598 | 9,587,836 |
|  | Blood glucose levels | [ebi-a-GCST90025986](https://gwas.mrcieu.ac.uk/datasets/ebi-a-GCST90025986/) | 400,458 | 4,218,897 |
|  |  |  |  |  |
| **Outcomes** |  |  |  |  |
|  | depression | [ukb-b-12064](https://gwas.mrcieu.ac.uk/datasets/ukb-b-12064/) | 462,933 | 9,851,867 |
|  |  |  | 482,730 | 17,891,936 |

|  |  |  |  | **TyG Index SNP Data** | | |  |  |  |  |
| --- | --- | --- | --- | --- | --- | --- | --- | --- | --- | --- |
| chr.exposure | pos.exposure | beta.exposure | se.exposure | pval.exposure | samplesize.exposure | id.exposure | SNP | effect_allele  exposure | other_allele  exposure | eaf.exposure |
| 18.00 | 40672964.00 | -0.01 | 0.00 | 0.00 | 273368.00 | tyg | rs7296326 | T | C | 0.33 |
| 18.00 | 40672964.00 | 0.01 | 0.00 | 0.00 | 273368.00 | tyg | rs2302883 | T | C | 0.33 |
| 18.00 | 40672964.00 | -0.01 | 0.00 | 0.00 | 273368.00 | tyg | rs10811661 | T | C | 0.33 |
| 18.00 | 40672964.00 | -0.03 | 0.01 | 0.00 | 273368.00 | tyg | rs147764624 | G | C | 0.33 |
| 2.00 | 169119609.00 | -0.01 | 0.00 | 0.00 | 273368.00 | tyg | rs213498 | T | A | 0.13 |
| 18.00 | 40672964.00 | 0.01 | 0.00 | 0.00 | 273368.00 | tyg | rs2250900 | C | T | 0.33 |
| 18.00 | 40672964.00 | 0.01 | 0.00 | 0.00 | 273368.00 | tyg | rs12173130 | T | C | 0.33 |
| 18.00 | 40672964.00 | 0.01 | 0.00 | 0.00 | 273368.00 | tyg | rs38205 | C | A | 0.33 |
| 18.00 | 40672964.00 | 0.01 | 0.00 | 0.00 | 273368.00 | tyg | rs13074711 | T | C | 0.33 |
| 18.00 | 40672964.00 | 0.01 | 0.00 | 0.00 | 273368.00 | tyg | rs73198299 | T | C | 0.33 |
| 18.00 | 40672964.00 | 0.03 | 0.00 | 0.00 | 273368.00 | tyg | rs115128825 | C | A | 0.33 |
| 18.00 | 40672964.00 | -0.01 | 0.00 | 0.00 | 273368.00 | tyg | rs4709746 | C | T | 0.33 |
| 18.00 | 40672964.00 | -0.02 | 0.00 | 0.00 | 273368.00 | tyg | rs34820917 | G | A | 0.33 |
| 18.00 | 40672964.00 | -0.01 | 0.00 | 0.00 | 273368.00 | tyg | rs6710938 | A | C | 0.33 |
| 18.00 | 40672964.00 | 0.01 | 0.00 | 0.00 | 273368.00 | tyg | rs2971676 | G | A | 0.33 |
| 18.00 | 40672964.00 | 0.01 | 0.00 | 0.00 | 273368.00 | tyg | rs151913 | G | A | 0.33 |
| 18.00 | 40672964.00 | 0.01 | 0.00 | 0.00 | 273368.00 | tyg | rs9935836 | A | C | 0.33 |
| 18.00 | 40672964.00 | -0.01 | 0.00 | 0.00 | 273368.00 | tyg | rs4134363 | G | A | 0.33 |
| 18.00 | 40672964.00 | -0.01 | 0.00 | 0.00 | 273368.00 | tyg | rs6066138 | G | A | 0.33 |
| 18.00 | 40672964.00 | 0.01 | 0.00 | 0.00 | 273368.00 | tyg | rs12885801 | C | A | 0.33 |
| 11.00 | 133764666.00 | 0.02 | 0.00 | 0.00 | 273368.00 | tyg | rs144470864 | A | C | 0.67 |
| 18.00 | 40672964.00 | 0.03 | 0.00 | 0.00 | 273368.00 | tyg | rs78025076 | C | T | 0.33 |
| 18.00 | 40672964.00 | 0.01 | 0.00 | 0.00 | 273368.00 | tyg | rs6437249 | C | T | 0.33 |
| 18.00 | 40672964.00 | -0.03 | 0.01 | 0.00 | 273368.00 | tyg | rs41290102 | C | T | 0.33 |
| 18.00 | 40672964.00 | 0.01 | 0.00 | 0.00 | 273368.00 | tyg | rs11781356 | T | A | 0.33 |
| 18.00 | 40672964.00 | 0.01 | 0.00 | 0.00 | 273368.00 | tyg | rs4812995 | T | C | 0.33 |
| 18.00 | 40672964.00 | -0.01 | 0.00 | 0.00 | 273368.00 | tyg | rs6760053 | C | G | 0.33 |
| 18.00 | 40672964.00 | -0.01 | 0.00 | 0.00 | 273368.00 | tyg | rs11657238 | G | A | 0.33 |
| 1.00 | 205656453.00 | -0.01 | 0.00 | 0.00 | 273368.00 | tyg | rs72904790 | T | C | 0.85 |
| 18.00 | 40672964.00 | 0.02 | 0.00 | 0.00 | 273368.00 | tyg | rs61904855 | C | A | 0.33 |
| 10.00 | 121410917.00 | 0.01 | 0.00 | 0.00 | 273368.00 | tyg | rs111585158 | C | T | 0.02 |
| 18.00 | 40672964.00 | -0.01 | 0.00 | 0.00 | 273368.00 | tyg | rs7175132 | A | G | 0.33 |
| 18.00 | 40672964.00 | 0.01 | 0.00 | 0.00 | 273368.00 | tyg | rs8092347 | A | G | 0.33 |
| 18.00 | 40672964.00 | 0.01 | 0.00 | 0.00 | 273368.00 | tyg | rs12937081 | A | G | 0.33 |
| 18.00 | 40672964.00 | 0.03 | 0.01 | 0.00 | 273368.00 | tyg | rs187217942 | G | A | 0.33 |
| 18.00 | 40672964.00 | 0.01 | 0.00 | 0.00 | 273368.00 | tyg | rs3808976 | A | G | 0.33 |
| 3.00 | 58218352.00 | 0.02 | 0.00 | 0.00 | 273368.00 | tyg | rs72669514 | C | T | 0.37 |
| 16.00 | 30923602.00 | 0.01 | 0.00 | 0.00 | 273368.00 | tyg | rs34921778 | A | G | 0.66 |
| 18.00 | 40672964.00 | 0.01 | 0.00 | 0.00 | 273368.00 | tyg | rs852424 | C | T | 0.33 |
| 18.00 | 40672964.00 | 0.01 | 0.00 | 0.00 | 273368.00 | tyg | rs1585705 | A | C | 0.33 |
| 18.00 | 40672964.00 | -0.02 | 0.00 | 0.00 | 273368.00 | tyg | rs11216122 | G | T | 0.33 |
| 18.00 | 40672964.00 | -0.02 | 0.00 | 0.00 | 273368.00 | tyg | rs62459110 | G | C | 0.33 |
| 18.00 | 40672964.00 | -0.01 | 0.00 | 0.00 | 273368.00 | tyg | rs77244849 | T | C | 0.33 |
| 18.00 | 40672964.00 | -0.01 | 0.00 | 0.00 | 273368.00 | tyg | rs72739147 | A | T | 0.33 |
| 4.00 | 951947.00 | -0.01 | 0.00 | 0.00 | 273368.00 | tyg | rs1760801 | G | A | 0.20 |
| 17.00 | 16035225.00 | -0.01 | 0.00 | 0.00 | 273368.00 | tyg | rs80216311 | C | T | 0.57 |
| 3.00 | 182760073.00 | 0.01 | 0.00 | 0.00 | 273368.00 | tyg | rs17656269 | C | T | 0.18 |
| 4.00 | 90666041.00 | 0.02 | 0.00 | 0.00 | 273368.00 | tyg | rs76172548 | A | C | 0.62 |
| 18.00 | 40672964.00 | -0.02 | 0.00 | 0.00 | 273368.00 | tyg | rs71352934 | A | C | 0.33 |
| 18.00 | 40672964.00 | -0.01 | 0.00 | 0.00 | 273368.00 | tyg | rs11006681 | G | A | 0.33 |
| 18.00 | 40672964.00 | 0.04 | 0.01 | 0.00 | 273368.00 | tyg | rs79429216 | G | A | 0.33 |
| 18.00 | 40672964.00 | 0.01 | 0.00 | 0.00 | 273368.00 | tyg | rs2745400 | G | A | 0.33 |
| 18.00 | 40672964.00 | -0.01 | 0.00 | 0.00 | 273368.00 | tyg | rs390556 | T | C | 0.33 |
| 18.00 | 40672964.00 | 0.01 | 0.00 | 0.00 | 273368.00 | tyg | rs2975424 | T | C | 0.33 |
| 18.00 | 40672964.00 | -0.01 | 0.00 | 0.00 | 273368.00 | tyg | rs62132802 | C | T | 0.33 |
| 18.00 | 40672964.00 | 0.01 | 0.00 | 0.00 | 273368.00 | tyg | rs71603401 | A | G | 0.33 |
| 18.00 | 40672964.00 | 0.01 | 0.00 | 0.00 | 273368.00 | tyg | rs8025505 | C | T | 0.33 |
| 18.00 | 40672964.00 | 0.01 | 0.00 | 0.00 | 273368.00 | tyg | rs678614 | C | A | 0.33 |
| 18.00 | 40672964.00 | 0.01 | 0.00 | 0.00 | 273368.00 | tyg | rs10783828 | G | A | 0.33 |
| 18.00 | 40672964.00 | 0.02 | 0.00 | 0.00 | 273368.00 | tyg | rs185139895 | G | A | 0.33 |
| 18.00 | 40672964.00 | 0.01 | 0.00 | 0.00 | 273368.00 | tyg | rs10861679 | T | C | 0.33 |
| 18.00 | 40672964.00 | 0.01 | 0.00 | 0.00 | 273368.00 | tyg | rs35477346 | T | C | 0.33 |
| 18.00 | 40672964.00 | -0.01 | 0.00 | 0.00 | 273368.00 | tyg | rs72801474 | G | A | 0.33 |
| 4.00 | 15737348.00 | -0.01 | 0.00 | 0.00 | 273368.00 | tyg | rs340836 | T | C | 0.55 |
| 18.00 | 40672964.00 | 0.01 | 0.00 | 0.00 | 273368.00 | tyg | rs9891030 | G | A | 0.33 |
| 18.00 | 40672964.00 | -0.01 | 0.00 | 0.00 | 273368.00 | tyg | rs10206462 | T | C | 0.33 |
| 18.00 | 40672964.00 | 0.01 | 0.00 | 0.00 | 273368.00 | tyg | rs3822076 | T | A | 0.33 |
| 18.00 | 40672964.00 | -0.01 | 0.00 | 0.00 | 273368.00 | tyg | rs2244278 | C | A | 0.33 |
| 18.00 | 40672964.00 | -0.01 | 0.00 | 0.00 | 273368.00 | tyg | rs3025053 | G | A | 0.33 |
| 18.00 | 40672964.00 | 0.01 | 0.00 | 0.00 | 273368.00 | tyg | rs17694506 | T | C | 0.33 |
| 18.00 | 40672964.00 | 0.02 | 0.00 | 0.00 | 273368.00 | tyg | rs11651957 | G | A | 0.33 |
| 18.00 | 40672964.00 | -0.01 | 0.00 | 0.00 | 273368.00 | tyg | rs197156 | A | G | 0.33 |
| 18.00 | 40672964.00 | -0.02 | 0.00 | 0.00 | 273368.00 | tyg | rs142164605 | T | A | 0.33 |
| 18.00 | 40672964.00 | -0.02 | 0.00 | 0.00 | 273368.00 | tyg | rs2035816 | A | G | 0.33 |
| 18.00 | 40672964.00 | 0.01 | 0.00 | 0.00 | 273368.00 | tyg | rs6090040 | C | A | 0.33 |
| 18.00 | 40672964.00 | -0.03 | 0.00 | 0.00 | 273368.00 | tyg | rs75662196 | G | C | 0.33 |
| 18.00 | 40672964.00 | -0.03 | 0.00 | 0.00 | 273368.00 | tyg | rs57295072 | G | C | 0.33 |
| 6.00 | 32561334.00 | -0.01 | 0.00 | 0.00 | 273368.00 | tyg | rs11118610 | A | C | 0.15 |
| 18.00 | 40672964.00 | -0.01 | 0.00 | 0.00 | 273368.00 | tyg | rs8028620 | T | C | 0.33 |
| 18.00 | 40672964.00 | -0.01 | 0.00 | 0.00 | 273368.00 | tyg | rs2792736 | A | T | 0.33 |
| 18.00 | 40672964.00 | -0.01 | 0.00 | 0.00 | 273368.00 | tyg | rs3750571 | C | A | 0.33 |
| 18.00 | 40672964.00 | -0.01 | 0.00 | 0.00 | 273368.00 | tyg | rs2277844 | A | G | 0.33 |
| 18.00 | 40672964.00 | -0.02 | 0.00 | 0.00 | 273368.00 | tyg | rs57192995 | G | C | 0.33 |
| 5.00 | 60345424.00 | 0.01 | 0.00 | 0.00 | 273368.00 | tyg | rs3120619 | G | A | 0.11 |
| 8.00 | 16697579.00 | -0.01 | 0.00 | 0.00 | 273368.00 | tyg | rs907866 | G | A | 0.28 |
| 18.00 | 40672964.00 | 0.04 | 0.01 | 0.00 | 273368.00 | tyg | rs2075294 | G | T | 0.33 |
| 18.00 | 40672964.00 | -0.01 | 0.00 | 0.00 | 273368.00 | tyg | rs11045171 | A | G | 0.33 |
| 18.00 | 40672964.00 | 0.01 | 0.00 | 0.00 | 273368.00 | tyg | rs6448429 | C | T | 0.33 |
| 18.00 | 40672964.00 | 0.01 | 0.00 | 0.00 | 273368.00 | tyg | rs4715317 | G | T | 0.33 |
| 18.00 | 40672964.00 | -0.02 | 0.00 | 0.00 | 273368.00 | tyg | rs72754154 | G | A | 0.33 |
| 18.00 | 40672964.00 | 0.01 | 0.00 | 0.00 | 273368.00 | tyg | rs4804413 | C | T | 0.33 |
| 18.00 | 40672964.00 | 0.02 | 0.00 | 0.00 | 273368.00 | tyg | rs541012177 | G | T | 0.33 |
| 4.00 | 77183300.00 | -0.02 | 0.00 | 0.00 | 273368.00 | tyg | rs16836630 | G | C | 0.21 |
| 18.00 | 40672964.00 | 0.01 | 0.00 | 0.00 | 273368.00 | tyg | rs12934528 | T | C | 0.33 |
| 18.00 | 40672964.00 | 0.01 | 0.00 | 0.00 | 273368.00 | tyg | rs231539 | C | T | 0.33 |
| 18.00 | 40672964.00 | 0.02 | 0.00 | 0.00 | 273368.00 | tyg | rs11216236 | C | T | 0.33 |
| 18.00 | 40672964.00 | -0.01 | 0.00 | 0.00 | 273368.00 | tyg | rs7703744 | C | G | 0.33 |
| 18.00 | 40672964.00 | 0.01 | 0.00 | 0.00 | 273368.00 | tyg | rs58895965 | C | A | 0.33 |
| 18.00 | 40672964.00 | 0.02 | 0.00 | 0.00 | 273368.00 | tyg | rs113344423 | G | A | 0.33 |
| 18.00 | 40672964.00 | -0.01 | 0.00 | 0.00 | 273368.00 | tyg | rs6923241 | C | T | 0.33 |
| 18.00 | 40672964.00 | -0.01 | 0.00 | 0.00 | 273368.00 | tyg | rs13289566 | C | T | 0.33 |
| 17.00 | 44095467.00 | 0.01 | 0.00 | 0.00 | 273368.00 | tyg | rs12617848 | C | T | 0.22 |
| 18.00 | 40672964.00 | 0.03 | 0.00 | 0.00 | 273368.00 | tyg | rs74444445 | T | C | 0.33 |
| 18.00 | 40672964.00 | -0.02 | 0.00 | 0.00 | 273368.00 | tyg | rs55730499 | C | T | 0.33 |
| 18.00 | 40672964.00 | 0.01 | 0.00 | 0.00 | 273368.00 | tyg | rs67981690 | A | G | 0.33 |
| 18.00 | 40672964.00 | -0.03 | 0.00 | 0.00 | 273368.00 | tyg | rs1801689 | A | C | 0.33 |
| 18.00 | 40672964.00 | 0.01 | 0.00 | 0.00 | 273368.00 | tyg | rs1035941 | G | A | 0.33 |
| 18.00 | 40672964.00 | 0.02 | 0.00 | 0.00 | 273368.00 | tyg | rs17092008 | C | T | 0.33 |
| 18.00 | 40672964.00 | 0.03 | 0.00 | 0.00 | 273368.00 | tyg | rs2207132 | G | A | 0.33 |
| 18.00 | 40672964.00 | -0.03 | 0.00 | 0.00 | 273368.00 | tyg | rs117805502 | C | T | 0.33 |
| 18.00 | 40672964.00 | 0.02 | 0.00 | 0.00 | 273368.00 | tyg | rs5880 | G | C | 0.33 |
| 18.00 | 40672964.00 | -0.02 | 0.00 | 0.00 | 273368.00 | tyg | rs5110 | C | A | 0.33 |
| 18.00 | 40672964.00 | 0.01 | 0.00 | 0.00 | 273368.00 | tyg | rs11636087 | T | C | 0.33 |
| 18.00 | 40672964.00 | -0.01 | 0.00 | 0.00 | 273368.00 | tyg | rs2106727 | G | A | 0.33 |
| 18.00 | 40672964.00 | 0.01 | 0.00 | 0.00 | 273368.00 | tyg | rs62102718 | A | T | 0.33 |
| 18.00 | 40672964.00 | -0.01 | 0.00 | 0.00 | 273368.00 | tyg | rs112740904 | T | G | 0.33 |
| 18.00 | 40672964.00 | 0.01 | 0.00 | 0.00 | 273368.00 | tyg | rs12610709 | G | A | 0.33 |
| 18.00 | 40672964.00 | 0.02 | 0.00 | 0.00 | 273368.00 | tyg | rs71525127 | C | G | 0.33 |
| 18.00 | 40672964.00 | -0.01 | 0.00 | 0.00 | 273368.00 | tyg | rs11558471 | A | G | 0.33 |
| 18.00 | 40672964.00 | 0.01 | 0.00 | 0.00 | 273368.00 | tyg | rs1534696 | A | C | 0.33 |
| 18.00 | 40672964.00 | 0.01 | 0.00 | 0.00 | 273368.00 | tyg | rs2963476 | A | G | 0.33 |
| 15.00 | 61993702.00 | 0.02 | 0.00 | 0.00 | 273368.00 | tyg | rs35750610 | T | C | 0.74 |
| 18.00 | 40672964.00 | -0.05 | 0.01 | 0.00 | 273368.00 | tyg | rs186696265 | C | T | 0.33 |
| 18.00 | 40672964.00 | 0.02 | 0.00 | 0.00 | 273368.00 | tyg | rs2894211 | C | A | 0.33 |
| 18.00 | 40672964.00 | 0.01 | 0.00 | 0.00 | 273368.00 | tyg | rs2081687 | C | T | 0.33 |
| 18.00 | 40672964.00 | 0.02 | 0.00 | 0.00 | 273368.00 | tyg | rs12208357 | C | T | 0.33 |
| 18.00 | 40672964.00 | 0.04 | 0.00 | 0.00 | 273368.00 | tyg | rs146390218 | A | G | 0.33 |
| 18.00 | 40672964.00 | -0.03 | 0.00 | 0.00 | 273368.00 | tyg | rs12721078 | C | A | 0.33 |
| 18.00 | 40672964.00 | -0.01 | 0.00 | 0.00 | 273368.00 | tyg | rs10832027 | A | G | 0.33 |
| 18.00 | 40672964.00 | 0.03 | 0.00 | 0.00 | 273368.00 | tyg | rs62271373 | T | A | 0.33 |
| 18.00 | 40672964.00 | 0.02 | 0.00 | 0.00 | 273368.00 | tyg | rs77009508 | A | G | 0.33 |
| 18.00 | 40672964.00 | 0.01 | 0.00 | 0.00 | 273368.00 | tyg | rs7758790 | T | C | 0.33 |
| 18.00 | 40672964.00 | -0.02 | 0.00 | 0.00 | 273368.00 | tyg | rs390802 | G | A | 0.33 |
| 18.00 | 40672964.00 | -0.01 | 0.00 | 0.00 | 273368.00 | tyg | rs419925 | G | C | 0.33 |
| 18.00 | 40672964.00 | 0.02 | 0.00 | 0.00 | 273368.00 | tyg | rs35169799 | C | T | 0.33 |
| 18.00 | 40672964.00 | -0.01 | 0.00 | 0.00 | 273368.00 | tyg | rs1882491 | T | C | 0.33 |
| 18.00 | 40672964.00 | -0.02 | 0.00 | 0.00 | 273368.00 | tyg | rs71480323 | G | A | 0.33 |
| 18.00 | 40672964.00 | -0.01 | 0.00 | 0.00 | 273368.00 | tyg | rs11075253 | C | A | 0.33 |
| 18.00 | 40672964.00 | 0.01 | 0.00 | 0.00 | 273368.00 | tyg | rs62521590 | T | G | 0.33 |
| 18.00 | 40672964.00 | 0.01 | 0.00 | 0.00 | 273368.00 | tyg | rs10786069 | T | C | 0.33 |
| 18.00 | 40672964.00 | 0.01 | 0.00 | 0.00 | 273368.00 | tyg | rs7140110 | T | C | 0.33 |
| 1.00 | 155135036.00 | 0.04 | 0.00 | 0.00 | 273368.00 | tyg | rs114165349 | G | C | 0.02 |
| 18.00 | 40672964.00 | 0.02 | 0.00 | 0.00 | 273368.00 | tyg | rs7821812 | G | C | 0.33 |
| 18.00 | 40672964.00 | 0.01 | 0.00 | 0.00 | 273368.00 | tyg | rs10260148 | C | T | 0.33 |
| 18.00 | 40672964.00 | -0.02 | 0.00 | 0.00 | 273368.00 | tyg | rs28550053 | A | G | 0.33 |
| 18.00 | 40672964.00 | 0.04 | 0.00 | 0.00 | 273368.00 | tyg | rs55697600 | A | G | 0.33 |
| 18.00 | 40672964.00 | -0.03 | 0.00 | 0.00 | 273368.00 | tyg | rs61737373 | G | A | 0.33 |
| 18.00 | 40672964.00 | 0.02 | 0.00 | 0.00 | 273368.00 | tyg | rs904009 | A | C | 0.33 |
| 18.00 | 40672964.00 | -0.01 | 0.00 | 0.00 | 273368.00 | tyg | rs61362984 | A | G | 0.33 |
| 18.00 | 40672964.00 | 0.01 | 0.00 | 0.00 | 273368.00 | tyg | rs1388941 | G | A | 0.33 |
| 18.00 | 40672964.00 | -0.02 | 0.00 | 0.00 | 273368.00 | tyg | rs4722551 | T | C | 0.33 |
| 18.00 | 40672964.00 | 0.04 | 0.00 | 0.00 | 273368.00 | tyg | rs17119701 | A | G | 0.33 |
| 18.00 | 40672964.00 | -0.06 | 0.01 | 0.00 | 273368.00 | tyg | rs188247550 | C | T | 0.33 |
| 18.00 | 40672964.00 | 0.02 | 0.00 | 0.00 | 273368.00 | tyg | rs2925979 | C | T | 0.33 |
| 18.00 | 40672964.00 | -0.01 | 0.00 | 0.00 | 273368.00 | tyg | rs1967685 | G | C | 0.33 |
| 18.00 | 40672964.00 | -0.02 | 0.00 | 0.00 | 273368.00 | tyg | rs1716407 | A | G | 0.33 |
| 18.00 | 40672964.00 | 0.02 | 0.00 | 0.00 | 273368.00 | tyg | rs632057 | G | T | 0.33 |
| 18.00 | 40672964.00 | 0.02 | 0.00 | 0.00 | 273368.00 | tyg | rs13108218 | G | A | 0.33 |
| 18.00 | 40672964.00 | -0.02 | 0.00 | 0.00 | 273368.00 | tyg | rs484066 | T | A | 0.33 |
| 18.00 | 40672964.00 | -0.02 | 0.00 | 0.00 | 273368.00 | tyg | rs79953491 | A | G | 0.33 |
| 18.00 | 40672964.00 | 0.02 | 0.00 | 0.00 | 273368.00 | tyg | rs1471251 | A | T | 0.33 |
| 12.00 | 40885549.00 | -0.03 | 0.00 | 0.00 | 273368.00 | tyg | rs76384951 | A | C | 0.02 |
| 18.00 | 40672964.00 | 0.02 | 0.00 | 0.00 | 273368.00 | tyg | rs4921914 | T | C | 0.33 |
| 18.00 | 40672964.00 | -0.02 | 0.00 | 0.00 | 273368.00 | tyg | rs11134475 | G | A | 0.33 |
| 18.00 | 40672964.00 | 0.04 | 0.00 | 0.00 | 273368.00 | tyg | rs799157 | C | T | 0.33 |
| 18.00 | 40672964.00 | 0.02 | 0.00 | 0.00 | 273368.00 | tyg | rs3936511 | A | G | 0.33 |
| 18.00 | 40672964.00 | -0.08 | 0.01 | 0.00 | 273368.00 | tyg | rs187929675 | C | T | 0.33 |
| 18.00 | 40672964.00 | -0.02 | 0.00 | 0.00 | 273368.00 | tyg | rs12446515 | C | T | 0.33 |
| 18.00 | 40672964.00 | 0.02 | 0.00 | 0.00 | 273368.00 | tyg | rs1532085 | G | A | 0.33 |
| 12.00 | 123326598.00 | -0.05 | 0.00 | 0.00 | 273368.00 | tyg | rs533617 | T | C | 0.36 |
| 18.00 | 40672964.00 | 0.02 | 0.00 | 0.00 | 273368.00 | tyg | rs878521 | G | A | 0.33 |
| 18.00 | 40672964.00 | 0.02 | 0.00 | 0.00 | 273368.00 | tyg | rs99780 | C | T | 0.33 |
| 18.00 | 40672964.00 | -0.04 | 0.00 | 0.00 | 273368.00 | tyg | rs11600380 | T | C | 0.33 |
| 18.00 | 40672964.00 | -0.02 | 0.00 | 0.00 | 273368.00 | tyg | rs2943645 | T | C | 0.33 |
| 18.00 | 40672964.00 | -0.05 | 0.00 | 0.00 | 273368.00 | tyg | rs75919952 | C | T | 0.33 |
| 7.00 | 23245569.00 | 0.02 | 0.00 | 0.00 | 273368.00 | tyg | rs4846922 | C | T | 0.39 |
| 18.00 | 40672964.00 | 0.03 | 0.00 | 0.00 | 273368.00 | tyg | rs261334 | C | G | 0.33 |
| 18.00 | 40672964.00 | 0.03 | 0.00 | 0.00 | 273368.00 | tyg | rs6073958 | T | C | 0.33 |
| 18.00 | 40672964.00 | 0.07 | 0.00 | 0.00 | 273368.00 | tyg | rs139974673 | T | C | 0.33 |
| 18.00 | 40672964.00 | 0.07 | 0.00 | 0.00 | 273368.00 | tyg | rs72836561 | C | T | 0.33 |
| 18.00 | 40672964.00 | 0.08 | 0.00 | 0.00 | 273368.00 | tyg | rs17091881 | T | C | 0.33 |
| 18.00 | 40672964.00 | -0.05 | 0.00 | 0.00 | 273368.00 | tyg | rs58542926 | C | T | 0.33 |
| 18.00 | 40672964.00 | 0.11 | 0.01 | 0.00 | 273368.00 | tyg | rs268 | A | G | 0.33 |
| 18.00 | 40672964.00 | -0.11 | 0.00 | 0.00 | 273368.00 | tyg | rs116843064 | G | A | 0.33 |
| 18.00 | 40672964.00 | 0.04 | 0.00 | 0.00 | 273368.00 | tyg | rs6547692 | A | G | 0.33 |
| 2.00 | 135537119.00 | -0.04 | 0.00 | 0.00 | 273368.00 | tyg | rs10889332 | C | T | 0.45 |
| 18.00 | 40672964.00 | 0.04 | 0.00 | 0.00 | 273368.00 | tyg | rs483082 | G | T | 0.33 |
| 18.00 | 40672964.00 | -0.06 | 0.00 | 0.00 | 273368.00 | tyg | rs17145750 | C | T | 0.33 |
| 18.00 | 40672964.00 | -0.04 | 0.00 | 0.00 | 273368.00 | tyg | rs17321515 | A | G | 0.33 |
| 18.00 | 40672964.00 | -0.10 | 0.00 | 0.00 | 273368.00 | tyg | rs117026536 | G | T | 0.33 |
| 18.00 | 40672964.00 | 0.12 | 0.00 | 0.00 | 273368.00 | tyg | rs7930786 | G | C | 0.33 |
| 18.00 | 40672964.00 | 0.11 | 0.00 | 0.00 | 273368.00 | tyg | rs56225305 | G | A | 0.33 |
