# Supplementary material for: Machine learning study on predicting depressive symptoms and genetic correlations in Parkinson’s disease
Source: Front Aging Neurosci. 2025 Apr 9;17:1584005. doi: 10.3389/fnagi.2025.1584005 (PMC12014618; doi:10.3389/fnagi.2025.1584005)
Supplement: Supplementary file 2 [file Table_2.DOCX]

Supplemental table 2. Comparison of predictive results for Parkinson's disease depression risk using multiple machine learning methods in two cohorts.

|  | Model | Accuracy | Precision | Recall | F1 Score | AUC |
| --- | --- | --- | --- | --- | --- | --- |
| Haizhu cohort | Logistic Regression | 0.81 | 0.79 | 0.78 | 0.78 | 0.78 |
|  | Random Forest | 0.85 | 0.84 | 0.83 | 0.83 | 0.79 |
|  | Support Vector Machine | 0.82 | 0.8 | 0.79 | 0.79 | 0.77 |
|  | XGBoost | 0.86 | 0.85 | 0.84 | 0.84 | 0.78 |
| Banan cohort | Logistic Regression | 0.8 | 0.78 | 0.77 | 0.77 | 0.76 |
|  | Random Forest | 0.84 | 0.83 | 0.82 | 0.82 | 0.75 |
|  | Support Vector Machine | 0.81 | 0.79 | 0.78 | 0.78 | 0.74 |
|  | XGBoost | 0.85 | 0.84 | 0.83 | 0.83 | 0.75 |
